# Systemic infection and microglia activation: a prospective postmortem study in sepsis patients

**DOI:** 10.1186/s12979-019-0158-7

**Published:** 2019-07-30

**Authors:** D. Westhoff, J. Y. Engelen-Lee, I. C. M. Hoogland, E. M. A. Aronica, D. J. van Westerloo, D. van de Beek, W. A. van Gool

**Affiliations:** 1 Department of Neurology, Amsterdam Neuroscience, Amsterdam University Medical Center, University of Amsterdam, Amsterdam, Netherlands; 2 Department of Intensive Care medicine, Leiden University Medical Center, Leiden, Netherlands; 3 Department of Neuropathology, Amsterdam University Medical Center, University of Amsterdam, Amsterdam, Netherlands; 4 Swammerdam Institute for Life Sciences, Center for Neuroscience, University of Amsterdam, Amsterdam, Netherlands

**Keywords:** Sepsis, Delirium, Microglia, Neuroinflammation, Sepsis associated encephalopathy

## Abstract

**Background:**

Systemic infection is associated with long-term cognitive deficits and functional decline. In this study we hypothesized that severe systemic inflammation leads to a neuroinflammatory response that is characterized by microglial activation, and that these effects might be more pronounced in patients using medication with anticholinergic side-effects.

**Methods:**

Based on the results of a pilot study in 8 patients, we assessed the number of MHC-II and CD-68 positive cells by immunohistochemistry and compared the number of microglia in specific brain regions of 16 well-characterized patients with septic shock and 15 controls.

**Results:**

In the pilot study, patients with sepsis tended to have higher density of MHC-II and CD-68 positive microglia in the basal ganglia (putamen, caudate nucleus and globus pallidus) and of MHC-II positive microglia in the hippocampus. In the validation study, patients with sepsis had a significantly higher number of CD-68 positive cells in hippocampus (1.5 fold; *p* = 0.012), putamen (2.2 fold; *p* = 0.008) and cerebellum (2.5 fold; *p* = 0.011) than control patients. The density of MHC-II positive microglia was similar between sepsis and control groups. There was no consistent correlation between microglia counts and anti-cholinergic activity drugs score.

**Conclusion:**

In patients who die during septic shock, severe systemic inflammation is accompanied by localized and strong upregulation of CD-68 positive microglia, but not of MHC-II positive microglia. We identified regional differences in the brain with increased microglial activation in putamen, hippocampus and cerebellum.

## Background

Over 70% of patients with severe systemic infection develop sepsis-associated encephalopathy (SAE), varying from mild delirium to coma [1]. Recent studies have shown that critically ill patients are prone to develop long-term cognitive deficits and functional decline [2]. No definitive mechanisms have been identified, although a more severe and longer duration of encephalopathy seems to contribute to more adverse cognitive outcomes [3].

The mechanisms affecting brain function during systemic inflammation remain to be elucidated. In the absence of encephalitis or meningitis, peripheral inflammation gives rise to the activation of the central immune system via several immune-to-brain communication pathways. It has been hypothesized that activation of microglia, the immune cells of the brain, is crucial for development of SAE [4]. Under normal circumstances the microglial response is tightly regulated but in old age, in neurodegenerative disease or during the use of anticholinergic drugs, microglia cells may escape this inhibitory reflex and become neurotoxic [5]. This might lead to a self-propelling neuroinflammatory reaction, which could account for the strong association between SAE and long-term cognitive impairment and even dementia [6].

Although animal experiments and several small studies in humans tend to support a role for neuroinflammatory mechanisms in SAE, the microglial response has been poorly characterized in patients with systemic inflammation [7]. In this study we hypothesized that severe peripheral inflammation leads to a neuroinflammatory response that is characterized by microglial activation. Additionally, we evaluated whether the use of medication with anticholinergic side-effects is associated with more pronounced microglial activation as a consequence of failing inhibitory control of the pro-inflammatory response in the brain. We compared brain tissue of well-characterized patients with septic shock with controls, and assessed the degree of microglia activation in different brain areas.

## Methods

### Patients and clinical data

From May 2011 to January 2015 all patients over 18 years old who died and whose relatives consented to brain autopsy in the Academic Medical Center in Amsterdam, the Netherlands, were assessed for inclusion. Patients were excluded if their medical history indicated neurodegenerative disease or any other recent serious intracranial pathology, such as meningitis or brain hemorrhage. In all cases written informed consent from the patients themselves or their relatives was available to use brain tissue for research purposes. Tissue was obtained and used in a manner compliant with the Declaration of Helsinki.

Demographic data, medical history and use of medication prior to hospital admission were collected. Detailed clinical information was obtained from patient’s health records and the electronic hospital information system. Anticholinergic properties of medication were determined according to two different anticholinergic scoring systems: the anticholinergic cognitive burden list (ACB) [8] and the anticholinergic risk scale (ARS) [9]. The ACB scale identifies the severity of anticholinergic negative effects on cognition of medications. Medications with serum anticholinergic activity or in vitro affinity to muscarinic receptors but with no known clinically relevant negative effects receive a score of 1. Drugs with established and clinically relevant anticholinergic effects are considered as definite anticholinergics and score 2 or 3. The ARS is designed to estimate to what extent an individual patient may be at risk of anticholinergic adverse effects. Medications are scaled 0 to 3 according to their anticholinergic potential: 0 limited or none; 1 moderate; 2 strong; and 3 very strong.

Chronic renal failure was defined as at least one symptom of kidney damage (albuminuria; urinary sediment abnormalities; electrolyte abnormalities due to tubular disorders; abnormalities detected by histology; structural abnormalities detected by imaging; history of kidney transplantation) and a glomerular filtration rate below 60 mL/min for > 3 months [10]. Acute kidney injury (AKI) was defined according to the RIFLE criteria [11]. Acute respiratory distress syndrome (ARDS) was scored according to the Berlin definition [12]. No division was made between mild, moderate and severe ARDS. The acute physiology and chronic health evaluation (APACHE-IV) and sequential organ failure assessment (SOFA) scores were collected of all patients admitted to the intensive care unit (ICU). The APACHE-IV is a method to predict hospital mortality among critically ill adults, using a multivariate logistic regression procedure [13]. Higher APACHE-IV scores are associated with higher probability of death. The SOFA, a scoring system to describe organ dysfunction, is composed of specific scores related to six different organ systems: the respiratory, cardiovascular, hepatic, coagulation and renal systems [14]. Higher scores indicate a more extensive organ failure. Medical interventions (e.g. resuscitation, surgery) and use of medication were documented. Antimicrobial drug use in the week before death was corrected for days of hospital stay and converted to units of defined daily doses, according to the classification of the World Health Organization [15]. Prothrombin time and plasma levels of sodium, potassium, urea, creatinine, lactate, glucose, bilirubin, bicarbonate, hemoglobin, C-reactive protein (CRP), thrombocytes and leukocytes in the week before death were recorded. All cultures of microorganisms in the week prior to death were collected and all clinical signs of infection were documented.

Patients were assigned to either the control group or the systemic inflammation group based on the presence or absence of clinical signs. Patients were considered as controls if they had no or only one sign of systemic inflammatory response syndrome (SIRS): temperature > 38 or < 36 °C, heart rate > 90/min, respiratory rate > 20/min, white blood cell count (WBC) > 12000 cells/mm^3^ or < 4000 cells/mm^3^ [16].

Patients were assigned to the severe sepsis group if they had at least two signs of SIRS (see above) due to infection, and at least one sign of organ dysfunction: lactate > 2 mmol/L, ARDS [12], AKI [11], platelet count < 100 000/mL and/or disseminated intravascular coagulation (DIC) [17].

Patients were assigned to the septic shock group if they had severe sepsis combined also with signs of shock, defined as a systemic mean blood pressure (MAP) < 60 mmHg after adequate fluid resuscitation, or the need for dopamine or (nor)epinephrine to retain a MAP of > 60 due to infection [18].

A small histochemical pilot study was performed first to select brain areas for further analysis in the whole cohort.

### Brain tissue, histochemistry and evaluation

Six brain areas of each patient were available: middle frontal gyrus, basal ganglia, hippocampus, mesencephalon, medulla oblongata and cerebellum.

Brain tissue was fixed in 4% formalin and embedded in paraffin. Five μm sections were mounted on StarFrost advanced adhesive slides (MLS, Menen, Belgium) and deparaffinized. Endogenous peroxidase was quenched by H2O2 in methanol for 20 min. Antigen was retrieved by heating slides in an autoclave at 120 °C for 10 min in citrate buffer (0.01 M, pH 6.0). For immunohistochemistry, sections were incubated with primary antibody for 1 h at room temperature. For visualization of microglia, we used antibodies against Major histocompatibility complex (MHC) class II (human leukocyte antigen (HLA)-DP, −DQ, −DR [1:100, monoclonal mouse, clone CR3/43, DAKO, Glostrup, Denmark]) and against cluster of differentiation 68 (CD-68 [1:200, monoclonal mouse, clone PG-M1, Dako]). The ready-to-use BrightVision Poly-HRP / peroxidase system (Immunologic, Duiven, the Netherlands) was used as secondary antibody and 3,3′-diaminobenzidine (Sigma-Aldrich, Zwijndrecht, the Netherlands) was used as chromogen. Sections were counterstained with hematoxylin (Klinipath BV, Duiven, the Netherlands).

Luxol fast blue – Periodic acid-Schiff – hematoxylin staining was used to discriminate between white and gray matter and was compared to microglial staining to select regions for analysis.

Sections were dehydrated and coverslipped with Pertex (Klinipath). All slides were scanned with a Ventana iScan HT slide scanner (Roche, Basel, Switzerland) at 20x magnification. One or two square millimeter of each digital image was selected, and microglia cell bodies were manually counted by two independent observers, both blinded for all clinical data: a neuropathologist (JYEL) and a PhD student (DW).

### Statistical analysis

Pilot study results were used for power analysis using nQuery advisor (version 7.0, Statistical Solutions, Cork, Ireland). Power was set to 80%, significance at 0.05 (two-sided) and the effect size was estimated based on pilot results.

All other statistics were performed using SPSS (SPSS for Windows, version 20, IBM Corporation, Armonk, NY, USA). Quantitative variables are presented as mean with standard deviation (SD) or median with interquartile range (IQR) depending on distribution. Continuous variables were tested with Mann-Whitney U tests or Student t-tests. Categorical variables were analyzed using Chi-Square or Fisher Exact tests. In case of multiple groups, testing was performed with one-way ANOVA or Kruskal-Wallis. Statistical significance was set to *p* ≤ 0.05. For visual presentation of quantitative results, GraphPad Prism was used (GraphPad Software, version 6.07, La Jolla, CA, USA).

## Results

A series of 115 patients met inclusion criteria, of whom 41 patients were subsequently excluded (Table 1), leaving 74 patients for analysis.

**Table 1:** Excluded patients (*N* = 42)

Reason for exclusion	n (%)
Intracranial pathology	22 (52.4)
Hemorrhage / infarction	7 (16.7)
Hepatic encephalopathy	7 (16.7)
Intracranial infection	4 (9.5)
Other	4 (9.5)
Insufficient clinical data	6 (14.3)
No informed consent	4 (9.5)
Not enough / different brain areas sampled	4 (9.5)
Other	6 (14.3)

Brain tissue of eight patients was used for the pilot study. MHC-II and CD-68 positive microglia counts were perfomed in the middle frontal gyrus, internal and external capsule, cerebellum, basal ganglia (caudate nucleus, putamen, globus pallidus) and hippocampus of five patients without systemic inflammation and three patients with severe sepsis (*n* = 2) or septic shock (*n* = 1). Patients with systemic inflammation tended to have higher MHC-II and CD-68 positive microglia numbers in basal ganglia and higher MHC-II positive microglia in hippocampus. Based on these results, basal ganglia and hippocampus were selected for analysis in the whole cohort. To evaluate the possibility of localized differences, we also assessed microglia numbers in cerebellum. A power calculation based on estimates of the different effect sizes in this pilot study, indicated that group sizes between 10 and 16 patients were required in the further analyses to confidently avoid a type II error.

The eigth patients included in the pilot study were excluded from all further analyses to avoid a sampling error. Sixteen patients with septic shock and 15 control patients were randomly selected from the database. Patient’s characteristics at admission were similar in both groups (Table 2), althouh patients in the control group more frequently suffered from cardiac failure (6 of 15 [40%] vs. 1 of 16 [6%); *p* = 0.037).

**Table 2:** Patient’s characteristics

	Control *N* = 15	Septic shock *N* = 16	*p*-value
Female n (%)	7 (46.7)	5 (31.3)	0.473
Age in years. Median (IQR)	70.1 (65.2–87.4)	68.6 (58.9–71.7)	0.119
Living independently. N (%)	13 (86.7)	16 (100.0)	0.226
N of chronic illnesses. Median (IQR)	3 (1–4)	2 (0–3)	0.060
Medical history			
Diabetes. N (%)	4 (26.7)	4 (25.0)	1.00
Hypertension. N (%)	11 (73.3)	10 (62.5)	0.704
Heartfailure (NYHA ≥II). N (%)	6 (40.0)	1 (6.3)	0.037
Cardiac other. N (%)	11 (73.3)	8 (50.0)	0.273
Peripheral vasculopathy. N (%)	2 (13.3)	0	0.226
Dyslipidemia. N (%)	6 (40.0)	2 (12.5)	0.113
CVA. N (%)	3 (20.0)	1 (6.3)	0.333
Chronic kidney disease (≥G3). N (%)	5 (33.3)	3 (18.8)	0.433
Livercirrhosis. N (%)	0	1 (6.3)	0.325
COPD (GOLD≥2). N (%)	0	2 (12.5)	0.333
Inflammatory disease. N (%)	3 (20.0)	1 (6.3)	0.704
Malignancy. N (%)	4 (26.7)	6 (37.5)	
Immune compromised. N (%)	0	0	
Alcohol or drug abuse. N (%)	1 (6.7)	1 (6.3)	1.00
N regular medication. Median (IQR)	7.5 (5.3–12.5)	6 (2.0–12.0)	0.223
Anticholinergic scores. Median (IQR)			
ACB score.	2.5 (1.0–3.0)	1.0 (0.0–2.8)	0.146
ARS score.	0 (0–0)	0 (0–0.75)	0.423

*ACB* anticholinergic cognitive burden list, *ARS* anticholinergic risk scale, *COPD* chronic obstructive pulmonary disease, *CVA* cerebrovascular accident, *NYHA* New York heart association

The majority of patients with systemic inflammation was admitted to the ICU, while patients without inflammation were mostly admitted to the cardiac care unit (Table 3). In the systemic inflammation group, the highest SOFA score in the first week of ICU admission was median 15 (IQR 11–18), and the mean APACHE score was 119.3 (SD 28.6).

**Table 3:** Characteristics during hospital stay

	Control *N* = 15	Sepsis *N* = 16	*p*-value
Hospital admission			
Reason for admission. N (%)			
Cardiac	9 (60.0)	2 (12.5)	
Infection	0	7 (43.8)	
Elective surgery	3 (20.0)	2 (12.5)	
Emergency surgery	0	1 (6.3)	
Other medical	3 (20.0)	4 (25.0)	
Ward. N (%)			
Cardiology	5 (33.3)	0	
Intensive care unit	2 (13.3)	15 (93.8)	
Emergency room	2 (13.3)	0	
Internal medicine	2 (13.3)	1 (6.3)	
Other	4 (26.6)	0	
Complications during hospital stay			
AKI. N (%)	1 (6.7)	10 (62.5)	< 0.001
ARDS. N (%)	1 (6.7)	7 (43.8)	0.013
Thrombocytopenia. N (%)	1 (6.7)	8 (50)	0.010
DIC. N (%)	0	12 (75)	< 0.001
Shock N (%)	10 (66.7)	16 (100)	0.018
*Septic*	*0*	*16 (100)*	
*Cardiogenic*	*8 (53.3)*	*0*	
*Obstructive*	*1 (6.7)*	*0*	
*Hypovolemic*	*1 (6.7)*	*0*	
Interventions during hospital stay			
Mechanical ventilation. N (%)	2 (13.3)	12 (75.0)	0.001
RRT. N (%)	0	7 (43.8)	0.007
Surgery. N (%)	3 (20.0)	7 (43.8)	0.152
Resuscitation. N (%)	9 (60.0)	6 (37.5)	0.186
Vasoactive medication	3 (20.0)	15 (81.3)	< 0.001
Infection and treatment			
At least 1 positive culture. N (%)	7 (46.7)	16 (100.0)	0.001
*Bacteremia*	*0*	*8 (50.0)*	
*Abdomen*	*0*	*8 (8.0)*	
*Respiratory tract*	*3 (20.0)*	*7 (43.8)*	
*Urogenital tract*	*4 (26.7)*	*2 (12.5)*	
*Peripheral*	*0*	*2 (12.5)*	
*Cardiac*	*0*	*1 (6.3)*	
N different microorganisms. Median (IQR)	0 (0.0–1.0)	1.5 (1.0–2.0)	0.003
Patients with antimicrobial therapy. N (%)	5 (33.3)	13 (81.3)	0.011
Defined daily doses. Median (IQR)	0 (0.0–0.7)	2.2 (1.0–3.3)	0.002
Death			
Cause of death. N (%)			
*Cardiac/hemodynamic*	14 (93.3)	2 (12.5)	< 0.001
*Respiratory*	*1 (6.7)*	*0*	
*MOF*	*0*	*13 (81.3)*	
*Other*	*0*	*1 (6.3)*	
Hospital stay, days. Median (IQR)	2.2 (1.2–8.2)	5.9 (2.1–14.6)	0.142
Active malignancy at death. N (%)	0	4 (25.0)	0.101
Hours between death and autopsy. Median (IQR)	21.7 (11.8–27.3)	18.4 (11.3–33.2)	0.608

*AKI* acute kidney injury, *ARDS* acute respiratory distress syndrome, *APACHE IV* acute physiology and chronic health evaluation, *DIS* disseminated intravascular coagulation, *MOF* multiple organ failure, *RRT* renal replacement therapy, *SOFA* sequential organ failure assessment

Forty percent of patients in the control group had a positive bacterial culture without clinical signs of inflammation. These infections were all in the respiratory or urinary tracts, and the majority was diagnosed at autopsy only. In the systemic inflammation group, all patients had at least one positive bacterial culture in the week before death. Consequently, the total antimicrobial use during hospital stay, as measured by defined daily doses (DDD), was higher in the systemic inflammation group than in the control group (median 2.2 vs 0 DDD, respectively, *p* = 0.002).

All patients with systemic inflammation developed septic shock in the week before death, while in the control group 10 patients (66%) suffered from shock, mostly cardiogenic shock (*p* = 0.018). Consequently more patients with systemic inflammation required vasoactive medication to retain a MAP of > 60 mmHg. They were more likely to have ARDS and/or AKI, and more frequently needed mechanical ventilation and renal replacement therapy than patients without signs of inflammation.

Patients with severe systemic inflammation had higher CRP levels [19] and they were more likely to exhibit leukopenia [20], thrombocytopenia [21] and DIC, than patients without systemic inflammation. Furthermore, they had higher mean arterial bicarbonate levels. Other blood components did not differ between groups.

Almost all control patients without signs of inflammation died of cardiopulmonary failure, while patients with systemic inflammation were more likely to die because of multiple organ dysfunction syndrome (MODS). The median time spent in the hospital before death was similar between groups as was the postmortem delay (Table 3).

To assess the agreement on microglia counts between observers, the differences between counts were plotted against the averages in Bland Altman plots. To correct for systematic differences between observers, all counts were normalized by dividing each count by the overall average count of the observer. Limits of agreement were +/− 0.68 for hippocampus; +/− 0.58 for caudatus; +/− 0.48 for globus pallidus; +/− 0.55 for putamen; and +/− 0.54 for cerebellum.

**Fig. 1:**
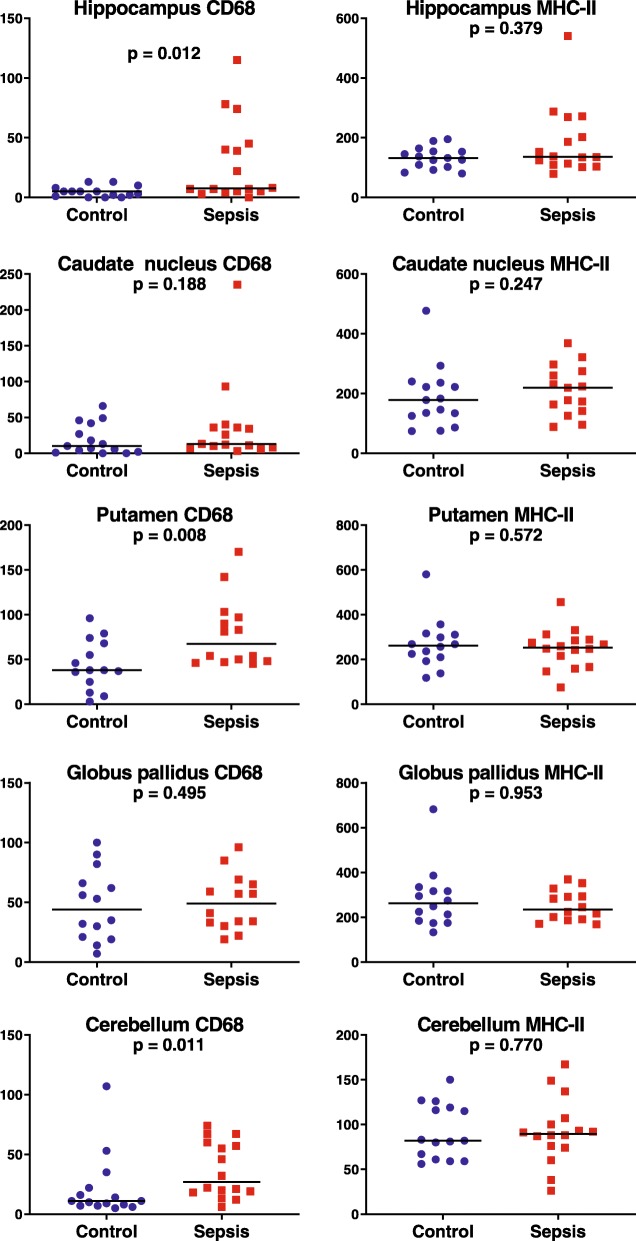
Numbers of CD68 and MHC-II positive microglia in hippocampus (CA1 region), basal ganglia and cerebellum

**Fig. 2:**
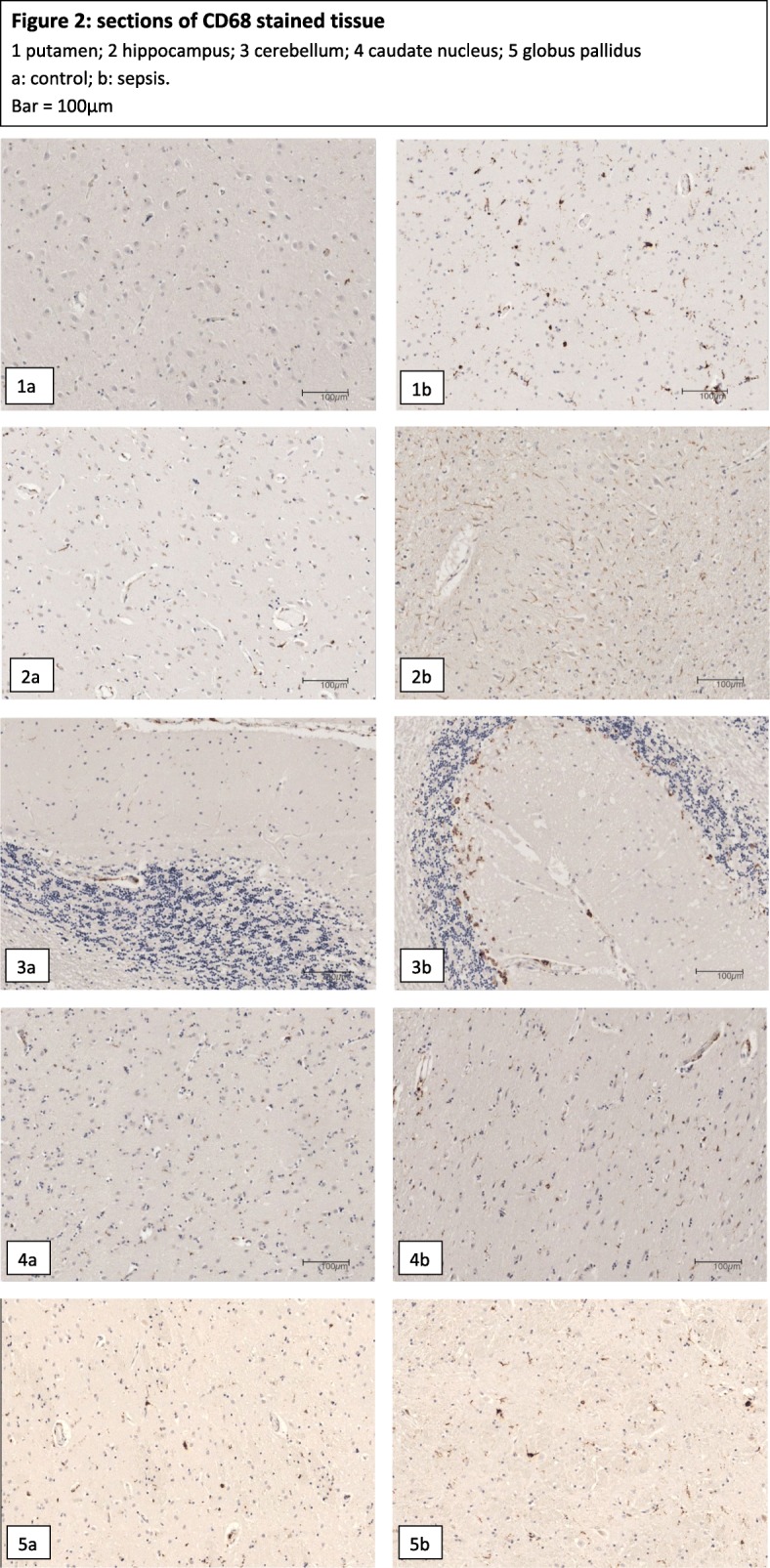
1–2.5 Sections of CD68 stained brain tissue. Microglia appear brown. 3.1 Hippocampus; 3.2 Putamen; 3.3 Caudate Nucleus; 3.4 Globus pallidus; 3.5 cerebellum. a: control, b: sepsis

MHC-II positive microglia numbers were comparable between patients with and without systemic inflammation (Table 4 and Fig. 1). Patients with systemic inflammation had significantly more CD-68 positive microglia in hippocampus, putamen and cerebellum than control patients without inflammation. Median counts in these areas were increased by 50, 116 and 145%, respectively. The higher CD-68 positive microglia counts in sepsis patients in the caudate nucleus and globus pallidus were not significantly different from those in controls. Representative sections of CD-68 stained slides in both sepsis and control patients are shown in Fig. 2.

**Table 4:** Number of microglia in different brain areas

	MHC-II staining			CD-68 staining		
	Sepsis	Control	*p*-value	Sepsis	Control	*p*-value
Hippocampus	136 (110–252)	132 (102–154)	0.379	7.5 (5–44)	5 (1–8)	0.012
Caudate nucleus	221 (146–291)	178 (125–235)	0.247	19.5 (9–39)	10 (2–42)	0.188
Globus pallidus	264 (193–346)	275 (184–335)	0.953	57 (33–81)	53 (21–82)	0.495
Putamen	253 (178–287)	268 (210–316)	0.572	82.0 (49–132)	38 (25–74)	0.008
Cerebellum	89 (74–105)	82 (61–119)	0.770	27.0 (18–59)	11 (7–22)	0.011

All data are presented as median with IQR

Variation in microglia numbers between patients was large (Fig. 1). To assess this variation we analyzed whether microglia activation was dependent on sex, age, medical history, length of hospital stay, or time between death and autopsy. We did not find a statistically relevant relationship between these factors and microglia numbers, neither when analyses were repeated in control patients separately.

To assess whether inhibition of microglia activation was affected by use of anticholinergic mediaction, we correlated microglia counts to two different anticholinergic scoring systems. None of the 30 correlations tested were significant for the ARS and counts of either MHC-II or CD-68 positive microglia in any brain region, not in the sepsis or control group separately, nor in both groups combined. The ACB scale significantly correlated with both MHC-II and CD-68 positive microglia counts in putamen of control patients (Spearman’s rho 0.617 and 0.602 respectively) and negative correlations were found with ACB scores and microglia counts in the hippocampus (Spearman’s rho − 0.394, all patients combined) and in the putamen (Spearman’s rho − 0.555 in sepsis patients).

## Discussion

In a prospective cohort study we assessed whether systemic inflammation leads to a neuroinflammatory response in the form of microglia activation. We found higher densities of CD-68 positive microglia in selected brain regions of patients with septic shock. This finding is suggestive of activation of the immune system in the brain, possibly explaining long term cognitive deficits in patients who survive severe systemic inflammation.

Two previous studies have been published that evaluate microglia activation through histopathology of the human brain. One case control study showed, consistent with the present findings, a significant increase in CD-68 expression in patients with sepsis compared to controls while no difference was seen in MHC-II staining [7]. Another study compared microglia activation in elderly patients with or without delirium at the time of death [22]. Patients who died during a delirious episode had more microglia activation as illustrated by upregulation of MHC-II and CD-68 positive cells. Yet this difference disappeared if patients with peripheral infection were excluded, consistent with the present results, suggesting that microglia activation is associated with systemic inflammation rather than delirium per se.

We found more CD-68 positive microglia in patients with systemic inflammation, while we found no difference in MHC-II positive microglia numbers. Markers that identify microglia in histochemistry are limited, and do not reliably signal specific functions or the activation state of microglia [23]. The most widely used microglia markers are MHC-II and CD-68. MHC-II is expressed by microglial cells with great morphological heterogeneity, irrespective of the level of activation [24]. It has been suggested that MHC-II is upregulated in ageing, and may not reflect (additional) upregulation in inflammation [25]. We assessed whether age is correlated with MHC-II positive cell numbers, but we did not find a statistical relevant association. CD-68 is a lysosomal membrane marker indicative of phagocytic activity [26]. In this study CD-68 microglia numbers differed between groups, whereas no differences were found in MHC-II cell numbers, possibly attributable to alteration of microglia function in the presence of severe systemic inflammation.

CD-68 microglia numbers were higher in putamen, cerebellum and hippocampus of patients with systemic inflammation, whereas groups did not significantly differ in microglia numbers in caudate nucleus and globus pallidus. Previous research showed that a single systemic challenge with lipopolysaccharide (LPS) significantly increased microglial proliferation in the hippocampus but not the cerebral cortex and corpus callosum of adult mice [27]. Future research might focus on explanations for these regional differences in microglia activation during systemic inflammation.

It has been suggested that the effects of acetylcholine might counteract microglia activation: an anti-inflammatory pathway by which the brain senses and modulates the systemic inflammatory response through the vagus nerve [6]. We assessed whether patients who used more anticholinergic medications prior to hospital admission, have a higher amount of neuroinflammation, illustrated by microglia activation. The gold standard for quantification of anticholinergic burden of medications would be measurement of serum anticholinergic activity. However, interpretation of the results in clinical practice is difficult. It has been suggested that serum anticholinergic activity might only assess peripheral activity, instead of central anticholinergic effects. Moreover, endogenous sources of anticholinergic activity in acute infection or stress contribute to the global anticholinergic burden [28]. One way to avoid these problems is the use of anticholinergic scoring systems. Since there is no widely accepted standard for such scoring, we explored two different scoring systems. The correlations between the ACB scale and microglia numbers in the putamen and hippocampus that we found need to be interpreted with caution, because of the explorative, multiple testing we performed. Moreover, not all medication used in the Netherlands is included in the anticholinergic scores. Furthermore, the two scales used do not agree on which medication should be included, and different scores are given to the same drug in each list [28].

Several alternative explanations for our results merit consideration such as the potential role of mechanical ventilation, the effects of shock or the influence of specific medication that was used. Mechanical ventilation might activate the immune system, causing damage to organs including the brain [29]. In our study, more patients in the sepsis group needed respiratory support than control patients, however, no association was found between both MHC-II and CD-68 microglia activation and mechanical ventilation. Furthermore, we only included patients with septic shock; one could argue that our results are attributable to shock rather than to inflammation. Yet more than half of patients in the control group suffered from shock, mostly cardiogenic. We did not find an association between microglia activation and shock per se.

Patients received selective decontamination of the digestive tract (SDD) if they were expected to be in the ICU for at least 3 days, or to need mechanical ventilation for at least 2 days [30]. The aim of SDD is to prevent nosocomial infections caused by potentially pathogenic microorganisms. Sixty-seven percent of sepsis patients received SDD, while in the control group no one received SDD. We cannot rule out that microglia activation was caused by SDD, however, it seems unlikely that addition of preventive antibiotics to a median of 2,2 DDD of therapeutic antibiotics could cause this significant difference. Moreover, we did not find a statistical relevant correlation between number of antibiotics and microglia activation.

We did not record previous episodes of inflammation or sepsis, possibly confounding our results; however, this applies to both groups. Time between death and brain autopsy was similar between groups, ruling out post mortem processes as an explanation for our results.

In this study we used brain tissue to evaluate neuroinflammation, where predictive serum biomarkers would offer a valuable tool. However, in our study the reliability of these markers is jeopardized by the immune response induced by systemic inflammation that may be indistinguishable from central nervous system inflammation. Future research focused on serum biomarkers would possibly increase our understanding of neuroinflammatory processes.

## Conclusion

In patients who die during septic shock, systemic inflammation is accompanied by localized and strong upregulation of CD-68 positive microglia, but not of MHC-II positive microglia. This finding is suggestive of activation of the immune system in the brain, possibly explaining long-term cognitive deficits in patients who survive severe systemic inflammation.

## Data Availability

The datasets used and/or analysed during the current study are available from the corresponding author on reasonable request.

## ABBREVATIONS

ACB: Anticholinergic cognitive burden
AKI: Acute kidney injury
APACHE-IV: Acute physiology and chronic health evaluation
ARDS: Acute respiratory distress syndrome
ARS: Anticholinergic risk scale
CD: Cluster of differentiation
COPD: Chronic obstructive pulmonary disease
CRP: C-reactive protein
CVA: Cerebrovascular accident
DIC: Disseminated intravascular coagulation
HLA: Human leukocyte antigen
ICU: Intensive care unit
MAP: Mean arterial pressure
MHC-II: Major histocompatibility complex class II
MODS: Multiple organ dysfunction syndrome
NYHA: New York Heart Association
RRT: Renal replacement therapy
SAE: Sepsis associated encephalopathy
SIRS: Systemic inflammatory response syndrome
SOFA: Sequential organ failure assessment
WBC: White blood cell count
